# Comparative Efficacy RCT of 3 Intensive Infant/Toddler Therapies for Unilateral Cerebral Palsy

**DOI:** 10.1542/pedsos.2025-000676

**Published:** 2026-02-20

**Authors:** Stephanie C. DeLuca, Sharon Landesman Ramey, Amy R. Darragh, Mark Conaway, Jill C. Heathcock, Warren Lo, Andrew M. Gordon, Mary Rebekah Trucks, Dory Wallace, Thais Invencao Cabral

**Affiliations:** 1 Fralin Biomedical Research Institute, Virginia Tech, Roanoke, Virginia; 2 Virginia Commonwealth University, Richmond, Virginia; 3 The University of Virginia, Charlottesville, Virginia; 4 The Ohio State University, Columbus, Ohio; 5 Nationwide Children’s Hospital, Columbus, Ohio; 6 Columbia University, New York, New York

## Abstract

**OBJECTIVES:**

Unilateral cerebral palsy (UCP) can result in lifelong upper extremity (UE) neuromotor impairment. While both constraint-induced movement therapy (CIMT) and bimanual training have demonstrated efficacy for children with UCP, there was limited evidence to inform treatment decision-making in children aged between 6 and 24 months. Thus, we performed a comparative efficacy trial testing 3 high-dose therapist-delivered interventions, 2 CIMT interventions varied by constraint type to bimanual/no-constraint intervention for use in treating this age group of children with UCP.

**PATIENTS AND METHODS:**

Fifty-eight infants/toddlers with UCP diagnosis, aged 6 to 24 months, were enrolled and randomized. Exclusion criteria were uncontrolled seizures, fragile health, prior CIMT/bimanual therapy, and recent botulinum toxin. Participants were randomly assigned (1:1:1) to 1 of 3 treatments all delivered 3 hours/d and 5 days/wk for 4 weeks: CIMT/full-time cast, CIMT/part-time splint, or bimanual/no constraint. Anonymized assessments at baseline, end of treatment (EoT), and 6 months posttreatment included the Mini–Assisting Hand Assessment (AHA) for bimanual abilities and the Bayley Scales of Infant and Toddler Development, Third Edition (Bayley-III) Fine-Motor (FM) subscale on each UE for FM abilities.

**RESULTS:**

Fifty-three infant/toddlers completed treatment and EoT assessment (mean age, 17.2 months), and 41 completed 6-month assessment. All groups had gains from intervention: Mini-AHA scores (*P* < .003) and Bayley-III FM/paretic side (*P* < .002). Bayley-III FM/nonparetic side also improved across groups (*P* < .001). The CIMT/full-time cast showed larger gains on Bayley-III FM/nonparetic side when compared with bimanual/no constraint (difference, 5.9; 95% CI, 1.2–10.5; *P* = .015).

**CONCLUSION:**

The trial confirms comparable benefits from therapist-delivered CIMT and bimanual/no-constraint interventions for infants/toddlers with UCP aged between 6 and 24 months.

## Introduction

Constraint-induced movement therapy (CIMT) and bimanual training are 2 rehabilitation interventions that have demonstrated the potential to ameliorate severe lifelong upper extremity (UE) neuromotor impairments associated with unilateral cerebral palsy (UCP).^1–11^ These interventions share similar key components. Both use reinforcement, shaping, and motor learning principles to help children achieve individualized UE goals and improve function with high levels of child engagement. What notably differentiates these approaches is that CIMT constrains the child’s less-impaired (nonparetic) UE during treatment, and bimanual training does not. While there are many versions of CIMT and bimanual training, the literature for both interventions is sufficiently supportive, such that, in 2021, Morgan et al^12^ issued a clinical practice guideline for infant/toddlers with UCP that endorsed CIMT and bimanual training. This recommendation was based on the demonstrated efficacy for these interventions in older children and in the growing literature surrounding infants/toddlers.^13–20^

However, clinicians and families often struggle to know which approach should be used for an individual child. A 2024 framework by Greeves and Hoar^21^ was designed to aid the decision-making process, but the authors recognized that the current literature has many gaps, stating that “While this paper aims to provide a comprehensive clinical framework for implementing upper limb therapy for infants with unilateral CP, we acknowledge that the current research evidence for upper limb therapy approaches for infants with unilateral CP less than 2 years of age remains limited.” Others in the field have agreed that more research is needed. Smith and Aravamuthan^22^ proposed that future studies “consider comparing high-dose CIMT to an equivalent dosage of therapy without restraint,” and Regalado et al’s^23^ review of 40 studies concluded there were “no differences in outcomes when bimanual approaches were compared with CIMT” but strongly advocated that “more Level 1b [(single, well-designed RCT)] studies are needed to compare CIMT with bimanual therapy to determine which one is most effective and under what conditions.” There are still crucial questions about intervention timing, individual patient responses, and how intervention characteristics might impact treatment response; furthermore, there is recognition that all of these issues likely interact.

For example, a recent trial by Boyd et al^15^ compared parent-conducted home-based CIMT and bimanual interventions in infants with or at risk for UCP. They found no between-group differences at short- or longer-term assessments, concluding that both interventions demonstrate benefits for infants in developing UE skills on the paretic side and in developing bimanual abilities. The study involved infants aged between 3 and 9 corrected months and stratified analyses between infants by those enrolled who were aged between 3 and 5 months and 6 and 9 months. Findings indicated that infants who enrolled in the earlier age group had greater changes. Timing mattered. Importantly, however, the older enrolled children also improved.

As stated earlier, there are many varied forms of CIMT and bimanual interventions reported in the literature, and, for infants, CIMT and bimanual training approaches are often parent delivered in part or in full. Most children with UCP receive a variety of therapist-delivered interventions across childhood, however. Thus, therapists and families need guidance about the potential differential benefits of such interventions. Baby CHAMP (Children with Hemiparesis Arm-and-Hand Movement Project) is an anonymized comparative efficacy trial designed to compare 3 high-dose interventions, all therapist-delivered: CIMT using 2 different forms of constraint, a full-time cast or a part-time splint, to bimanual, with no-constraint therapy. We hypothesized the following:

Hypothesis 1: All 3 treatments will improve both bimanual and fine-motor (FM) performance of the paretic UE. This is based on strong literature in support for both treatments.

Hypothesis 2: Bimanual/no-constraint therapy will yield better improvement in bimanual performance than CIMT because bimanual training permits learning and practice with both UEs.^8,21^

Hypothesis 3: CIMT/full-time cast will lead to greater gains in FM skills on the paretic UE compared with CIMT/part-time splint or bimanual/no constraint because wearing a cast full time strongly motivates using the paretic UE while reducing competing sensory-motor input from the nonparetic UE.^6,24^

Hypothesis 4: Constraint is not harmful. This is an important concern often raised by clinicians and parents. Could use of a constraint impair the child’s nonparetic UE, especially when used in infants and toddlers?^25,26^

## Methods

### Study Design

A multisite comparative efficacy randomized controlled trial (RCT) with allocation concealment and single anonymization (assessors were anonymized to group assignment) (clinicaltrials.gov registration NCT0234682) was approved by participating institutional review boards at Virginia Polytechnic Institute and State University and The Ohio State University; parents signed permission for child participation. Enrollment occurred January 2015 through December 2018.

### Participants

We recruited from local clinics, early intervention programs, and parent-advocacy websites and via parent-to-parent referrals. Inclusion criteria were UCP with a hemiparesis diagnosis, age of 6 to 24 months, and good health. Exclusion criteria were uncontrolled seizures, prior CIMT or bimanual training of more than 2 hours/d for longer than 9 days or botulinum toxin within 6 months.

### Randomization

The off-site study statistician conducted centralized randomization involving site stratification with 1:1:1 assignment to 3 groups using a random permuted block design (blocks, 6). The central study coordinator maintained a secure computer-generated randomization list; after parents consented, they learned their child’s group assignment and then scheduled a convenient treatment month.

### Procedures and Description of Treatment Interventions

Eight licensed pediatric occupational or physical therapists were centrally trained and certified competent to deliver all the multicomponent therapeutic protocol used for all 3 groups. Table 1 summarizes key therapy components across groups, incorporating those from the ACQUIRE^27–29^ signature form of CIMT tested in the CHAMP trial with 2- to 8-year-olds^6^ and compatible with published descriptions of hand-arm bimanual intensive therapy (HABIT) therapy, a signature form of bimanual training.^30^ Therapists continuously provide well-timed, response-contingent reinforcement to promote neuromotor progression in goal-directed activities, including play and self-help activities in a home/homelike setting. Parents supplement formal sessions with approximately 45 minutes/d of guided home practice. The dosage for all groups is 3-hour therapist-delivered sessions for 5 days/wk over 4 consecutive weeks (60 hours). Therapy key components, as described in Table 1, were consistent across groups with the exception of the constraint—whether constraint was used (eg, bimanual/no constraint or CIMT) and, if used, the constraint type (eg, cast or splint) and when it is worn (full time or part time) (see Supplemental Figure 1). All treatment protocols across groups included unilateral, bilateral, and whole-body activities. However, we acknowledge that there are practical differences in therapy delivery based on the use of constraint (ie, greater limitations in choices of bimanual activities in the CIMT groups.). For both CIMT groups, the constraint was removed entirely for the last 3 treatment days. For participants in the CIMT/full-time cast group, the therapist removed the cast for approximately 30 minutes each week to monitor skin integrity and allow movement of the nonparetic UE.

### Outcome Measures

Study-trained assessors anonymized to group assignment administered assessments at baseline (within 3 days pretreatment), end of treatment (EoT; within 3 days after), and 6 months posttreatment (within 2 weeks). Assessors had no contact with therapists or families outside assessment sessions, and families were instructed not to mention anything about treatment.

The primary outcome was the Mini–Assisting Hand Assessment (AHA)^31^ that assesses the use of the paretic UE during bimanual play from videotaped sessions by a single, anonymized Mini-AHA certified rater. Scoring occurred after all data collection, with session order concealed. The Mini-AHA was used for all children, even those aged more than 18 months, to ensure consistent administration and coding; other trials have also used the Mini-AHA is this manner.^18,32^ At the time of trial, comparability of scores from different age versions of this tool was not available. A recommended modification of the Bayley Scales of Infant and Toddler Development, Third Edtion (Bayley-III)^33^ FM subscale was a secondary outcome and provided separate FM scores for both the paretic/more-impaired and the nonparetic/less-impaired UE. The Bayley-III has strong psychometric properties and assesses development up to 42 months of age. This modification for scoring the FM subscale has been used in other rehabilitation trials.^13,19,34^ Exploratory outcomes included motor activities observed by parents, assessed using the Infant Motor Activity Log (IMAL). Parents completed the IMAL for “How Often” and “How Well” their child used the paretic UE in 20 age-appropriate activities.^13,34^ Parents also completed a survey about their child’s adaptation to constraint, if used, to determine possible differences in stress and frustration related to constraint type. Therapists maintained treatment logs about each session’s dosage, therapy goals and activities, child responses, any difficulties, and unanticipated or possible adverse events (AEs).

### Sample Size Determination

Sample size was determined to have sufficient power for between-group differences focused on bimanual changes, relying on an RCT with AHA scores for 2- to 8-year-olds in which large changes in AHA scores were observed.^10^ For large between-treatment effects (*η*^2^ = 0.15), the F-test has 80% power, with a significance level of 5%, with 20 children per group^35^ (see Supplement Table 1).

### Statistical Analyses

Analyses used SAS 9.4 and GAUSS 25.0 with CIs of 95% and/or *P* < .05 significance levels on the intent-to-treat sample. Repeated measures analyses of covariance, covarying for clinical site and baseline scores, was the primary method of analysis for comparing the 3 groups at EoT and 6 months posttreatment. Sensitivity analyses added sex and age as covariates to the repeated measures models. To check the sensitivity of conclusions to missing data, we applied random multiple imputation methods using regression imputation with 100 completed data sets.^36,37^

### Treatment Adherence, AEs, and Harm

Treatment adherence was defined as receiving the intended therapy dose, correctly wearing the constraint, adhering to treatment protocols based on weekly video-recorded sessions (ie, treatment fidelity), and active parent home program participation. AEs were documented via therapy notes; reported by parents, therapists, assessors, and study coordinators; and monitored by the site principal investigator. When an AE occurred, it was reported to a medical monitor and a data safety and monitoring committee. Constraint-related harm was assessed via AE reports and assessment of motor performance of the nonparetic UE at all time points using the Bayley-III FM/nonparetic side.

## Results

Sample characteristics are provided in Table 2 and Figure 1, showing that 55 children completed baseline assessment, 53 received assigned treatment plus EoT assessment (8.6% attrition from enrollment), and 41 completed 6-month assessment (29.4% attrition). We note this high attrition rate at 6 months particularly as a study limitation.

The sample mean age was 17.2 (SD, 4.8) months, with UCP diagnosis typically occurring between 4 and 8 months. Right-sided hemiparesis/UCP was the most prevalent (75%). Infant stroke was the most frequent parent-reported etiology. Mean birthweight and gestational age were within normal range (ie, >2500 g and >37 weeks, respectively). Children received a parent-reported average weekly therapy dose of 1.4 to 2.07 hours across groups (physical and occupational therapy) before the start of treatment in this trial.

Children in all 3 groups improved above baseline in their Mini-AHA scores, the primary outcome, at EoT and 6 months posttreatment, supporting hypothesis 1. Figures 2 and 3 show, numerically and graphically, the mean gains and 95% CIs within each group at each posttreatment time, along with pairwise comparisons between groups (see Supplemental Table 2 for summary values for the primary and secondary outcomes; the analysis of variance tables are listed in Supplemental Table 3). There were also gains over baseline at 6 months for the Bayley-III FM/paretic UE and FM/nonparetic UE outcomes. Smaller changes from baseline were observed on these outcomes for all 3 groups at the EoT except for the FM/nonparetic UE changes in the CIMT/part-time splint group, which had a 2.1-point improvement over baseline (95% CI, 0.1–4.1).

Regarding pairwise comparisons, hypotheses 2 and 3 are not supported because bimanual/no constraint did not produce significantly larger greater in bimanual outcomes, and CIMT/full-time cast did not yield higher FM gains, respectively. There were larger 6-month posttreatment gains for the CIMT/full-time cast group compared with bimanual/no constraint (difference, 5.9; 95% CI, 1.2–10.5) on the Bayley-III FM/nonparetic side. Hypothesis 4 is supported because the nonparetic UE maintained or gained function at both the EoT and at 6 months. Notably, this was the only group by time significant interaction on any outcome measure (ie, the CIMT/full-time cast group was significantly greater than the bimanual/ no constraint group at the 6 month post assessment).

Additional analyses that added covariates for sex and age at treatment in the repeated measures models (see Supplemental Figure 2) and assessed the effect of the missing data using multiple imputation (see Supplemental Figure 3) yielded nearly identical results. Mean IMAL ratings for “How often” and “How well” the child used the paretic/more-impaired UE increased above baseline significantly and comparably for all groups at EoT and 6 months posttreatment (*P* ≤ .001). In the constraint survey, 5 parents (26%) reported some short-term difficulties with the CIMT/full-time cast, and 3 parents (15%) reported short-term difficulties with the CIMT/part-time splint. Therapists’ logs indicated 77% of the children wearing the part-time splint showed splint-related frustration on at least 1 session, while 50% did with the full-time cast.

Treatment adherence included parent home program participation of more than 95% for each group. The 53 children who completed treatment all received the ±15 minutes of expected daily dosage. Weekly video recording scoring confirmed that therapists delivered treatment activities using motor learning and shaping progression for more than 80% of activities. Five AEs occurred, all deemed unrelated to treatment. These were hospitalization related to a prior surgery. A child in the bimanual/no-constraint group did not complete treatment. Three infants had onset of seizures—2 in the part-time splint group and 1 in the no-constraint group. One child developed dehydration caused by a stomach illness in full-time cast group. One child dropped from the part-time splint group because of a non–treatment-related family emergency.

## Discussion

Six- to 24-month-olds with UCP who received bimanual therapy or CIMT with full-time cast or part-time splint showed significant improvement in using their UEs. All received the same high dose of 5 therapist-delivered 3-hour sessions per week for 4 weeks. These findings support and extend the previous expert recommendations that CIMT and bimanual interventions yield highly comparable benefits. Ultimately, the findings upheld our first hypothesis that children would show improvements in all 3 groups. Our second and third hypotheses were not confirmed: We did not find group differences on bimanual or in unilateral FM performance. Regarding our fourth hypothesis, constraint did not appear to cause harm or reduce FM performance of the nonparetic UE. We note, however, that some parents and therapists reported constraints as disruptive, and skin irritation occurred in 10% of the children who wore a cast. All skin irritations were easily treated.

Our results align with other recent trials. Carton de Tournai et al^18^ found that 50 hours of a new form of infant HABIT including lower extremities produced gains in bimanual performance. Boyd et al^15^ found that both baby CIMT and baby bimanual treatments delivered by parents aided children in gaining unilateral and bimanual skills. Eliasson et al^20^ has provided evidence that approximately 36 hours of infant CIMT aids infants in the development of UE skills. Chamudot et al^17^ found benefits from parent-delivered CIMT and bimanual therapy in early childhood, and, particularly relevant to real-world gains in UE skills, Nordstrand et al^16^ found that early CIMT lead to improved UE abilities later in childhood.

In Baby CHAMP, we also saw significantly larger FM skill gains on the nonparetic UE for CIMT/full-time cast compared with bimanual/no constraint. This finding was unexpected but not unprecedented. Other trials, including some listed above, have shown improvements in the nonparetic UE after the use of a constraint. For example, an RCT by Maitre et al^19^ reported a similar change on the constrained, nonparetic UE for infants who received a multicomponent, parent-delivered form of CIMT in which infants wore a soft mitt 6 hours/d. Collectively, these findings support a long-standing recommendation from Basu and Eyre and others that studies of children with UCP should measure performance of both UEs.^38^ and others that studies of children with UCP should measure performance of both UEs. In fact, future research would benefit from measurements that routinely consider multidomains of development as well as longitudinal measurements of capacity and real-world use of both UEs. The field also is in need of ethnographic studies to document types and amounts of therapies received across development while simultaneously documenting patient and family characteristics. This detailed documentation would fill critical gaps in knowledge about how variations in rehabilitation treatments impact development across time by allowing multifactor analyses and predictive modeling and inform the most clinically beneficial rehabilitation pathways for children with UCP across time.

## Study Limitations

Baby CHAMP has notable limitations. First, sample size was 58 at enrollment with high attrition across assessment points. The reduction in power due to attrition may have increased the chances of a type II error. So, there is a possibility there may be differences that we did not detect because of levels of attrition; resultantly, we may need greater caution in interpreting our findings. For example, particular interpretation caution may surround our unpredicted finding at 6-month posttreatment indicating improvements in FM skills on the nonparetic UE in the casted group. Second, our analyses, also, made no formal adjustments for multiple comparisons, which further supports cautious interpretation of any findings. Third, the bimanual/no-constraint group had noticeably higher pretreatment scores at baseline on the primary outcome measure, something that can and does occur even with random assignment. We addressed this by following expert recommendations to covary for baseline scores. Thus, statistically, our findings indicate there are no differences on our primary outcome between groups. That said, this statistical correction does not negate the fact that the no-constraint/bimanual group was more functional at baseline. This real difference may have limited our ability to detect group differences. Fourth, between EoT and 6-month posttreatment, we recognize that multiple factors (such as family home practice or other medical treatments) could have influenced children’s performance; thus, our call for longitudinal ethnographic studies. Fifth, our primary outcome measure, the Mini-AHA, was used outside the recommended age range; while we did this for assessment consistency, we recognize that it may have provided an incomplete picture of bimanual abilities of children aged older than 18 months. Finally, Baby CHAMP lacked a conventional control/usual-treatment group. This decision was guided by the principle of equipoise based on evidence from many systematic reviews that deemed CIMT and bimanual therapies more efficacious than usual therapies. On reflection, our findings may be of most service to the field by being considered hypothesis generating vs an efficacy confirmatory study.

## Conclusions

This study provides evidence that high-dose therapist-delivered CIMT and bimanual training are both acceptable and safe for infants/toddlers (6 months to <2 years) with UCP. Accordingly, clinical relevance, family preference, and treatment goals should guide intervention choice in this age group. Parents and therapists may prefer bimanual training or CIMT for a range of reasons, and both interventions may be useful at different points in a child’s development.

## Supplementary Material

Supplemental 2

Supplemental 3

Supplemental 1

Supplemental 4

## Figures and Tables

**FIGURE 1.**
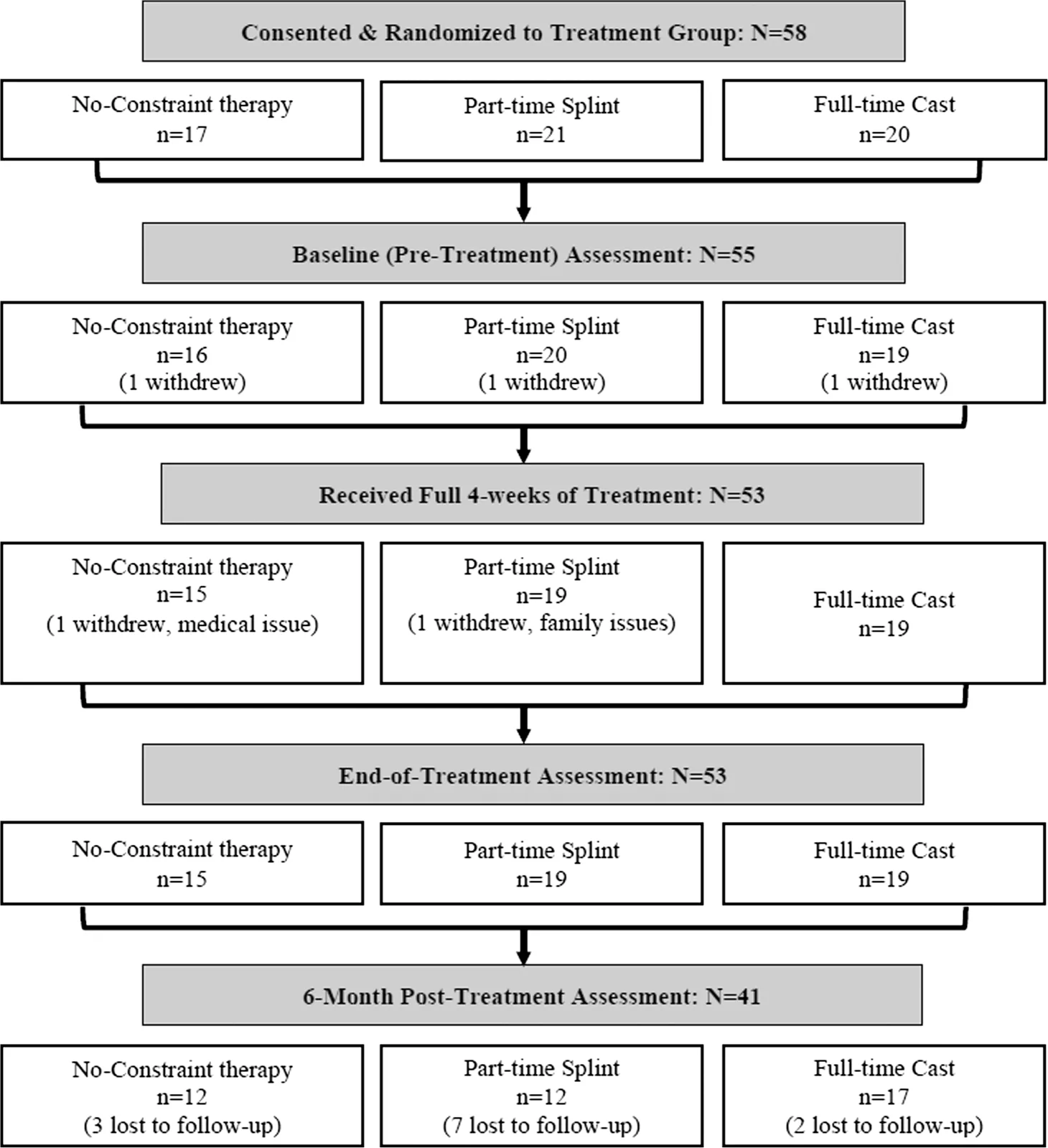
CONSORT diagram for Baby CHAMP. Abbreviation: CHAMP, Children with Hemiparesis Arm-and-Hand Movement Project.

**FIGURE 2.**
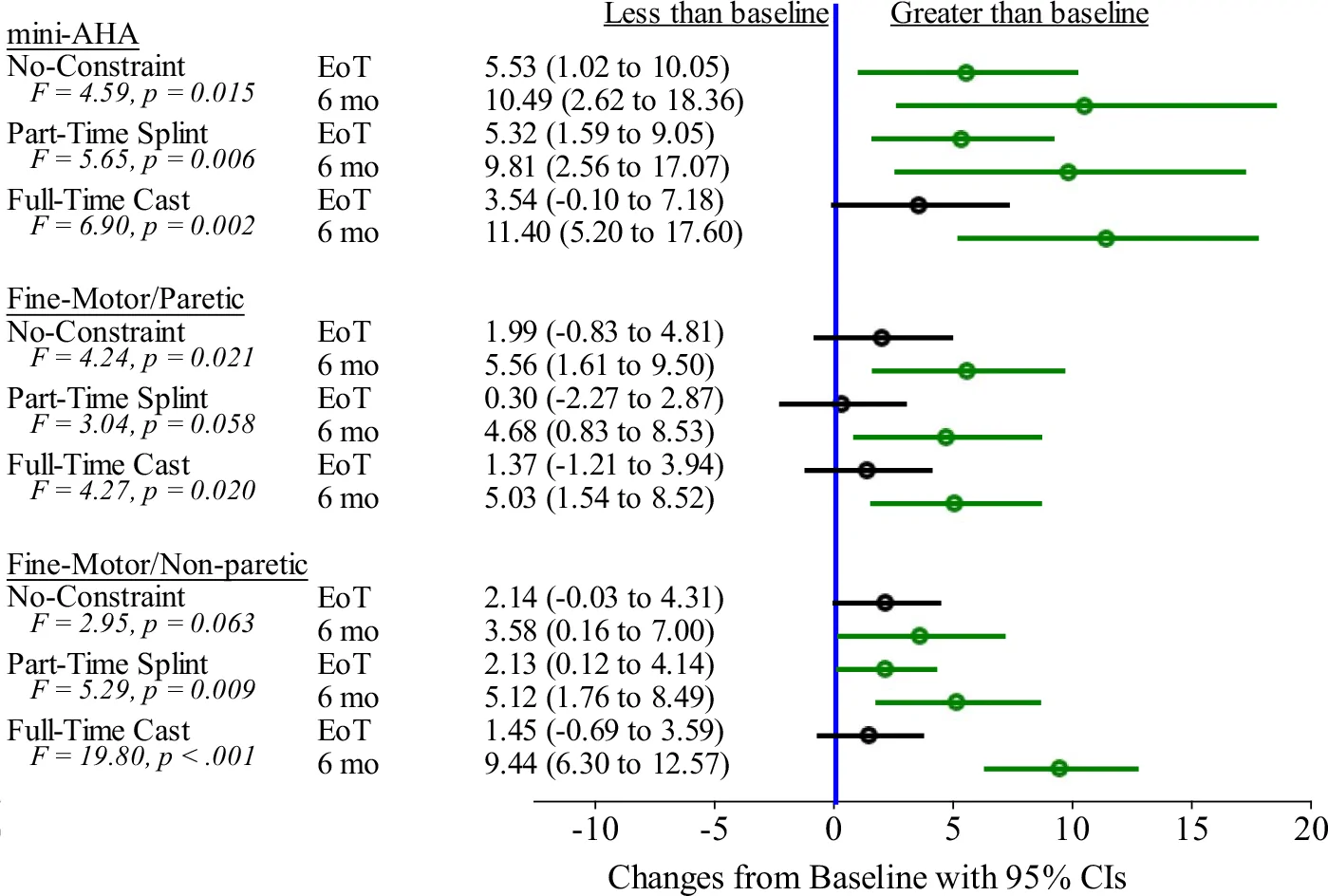
Within-group changes from baseline scores on major outcome measures (green lines indicate statistical significance). Dot in middle of each horizontal line represents estimated mean change score at each assessment point; placement on the *x*-axis represents direction and amount of group differences in points gained. Horizontal solid lines indicate 95% CIs. When CI lines fall entirely outside the blue vertical line (indicating zero changes from baseline), this denotes a statistically significant group difference—that is, the findings are likely to be replicated 95% of the time in similarly conducted clinical trials with this patient population (*P* < .05). Abbreviations: AHA, Assisting Hand Assessment; EoT, end of treatment.

**FIGURE 3.**
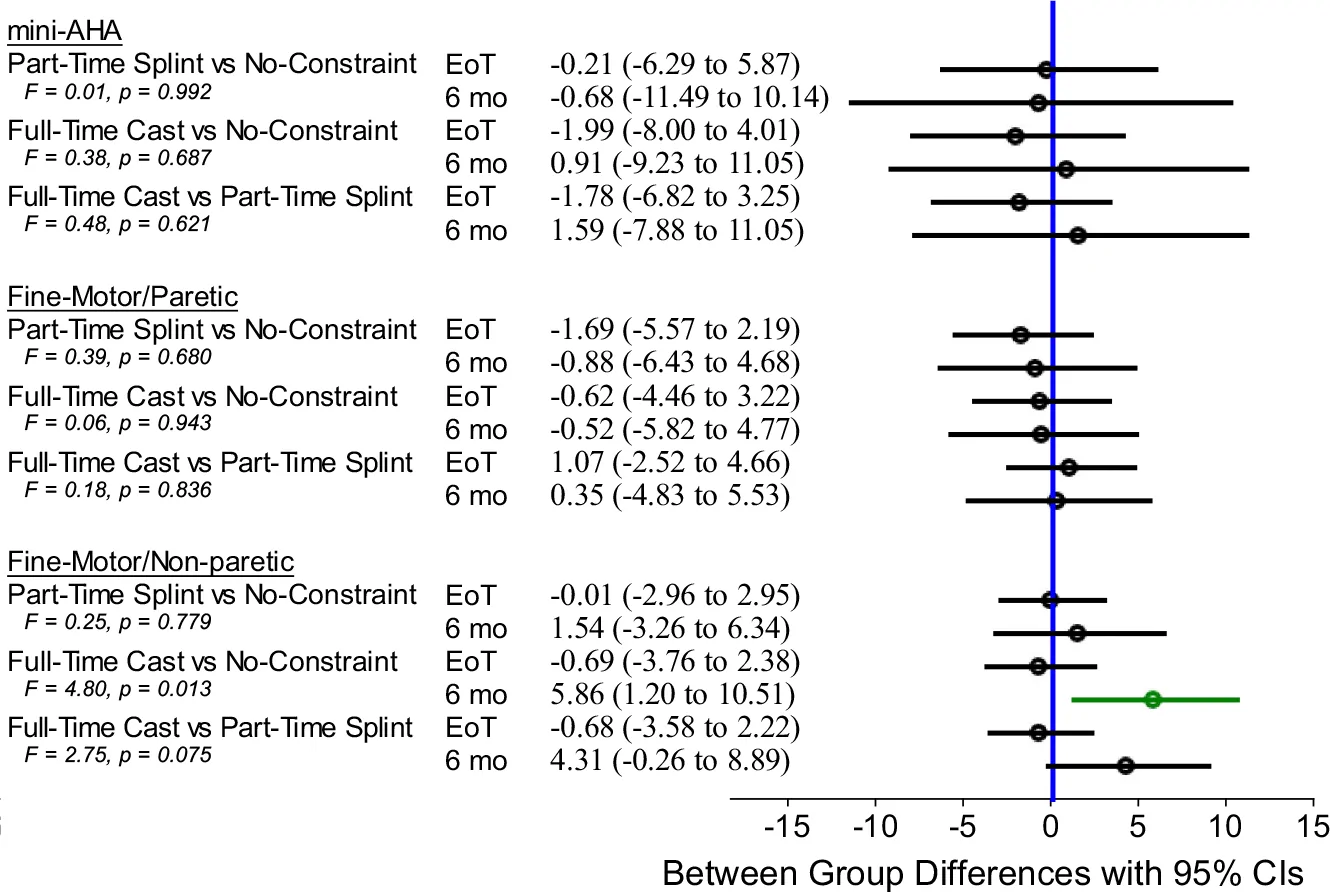
Between-group change scores on major outcomes at posttreatment assessments (green lines indicate statistical significance). Dot in middle of each horizontal line represents estimated mean difference in gain scores between the 2 compared groups (ie, mean change score of the first group minus that of the second group); placement on the *x*-axis represents direction and amount of group differences in points gained. Horizontal solid lines indicate 95% CIs. When CI lines fall entirely outside the blue vertical line (indicating zero group differences), this denotes a statistically significant group difference—that is, the findings are likely to be replicated 95% of the time in similarly conducted clinical trials with this patient population (*P* < .05). Abbreviation: EoT, end of treatment.

**TABLE 1.** Multiple Treatment Components for the 3 Treatment Groups in Baby CHAMP

Group 1 CIMT With Full-Time Cast	Group 2 CIMT With Part-Time Splint	Group 3 Bimanual/No Constraint

Continuous wearing of full-arm lightweight cast for first 17 of 20 therapy sessions. Constraint removed for final 3 d when therapy focus is on integrating new UE skills into bimanual activities.	Part-time use of part-arm splint worn only during first 17 therapy sessions. No constraint in final 3 d when therapy focus is on integrating new UE skills into bimanual activities.	No constraint is used during and in between formal treatment sessions.

**Treatment Components Common Across Groups**		

Dosage (relatively high): 20 three-h sessions, 5 d/wk, for 4 consecutive wk (total, 60 h).		
Therapy techniques (primarily operant conditioning: eg, reinforcement, shaping thorough successive approximations, varied practice opportunities): shaping with immediate reinforcement cycle (MR3 cycle), massed practice with planned variation, application of motor learning principles to progress skills and functional activities. Licensed pediatric OT/PTs are formally trained using the ACQUIRE manual and supplemental training materials throughout training; therapists must demonstrate understanding of all treatment components and display these later in videotaped sessions (see Supplemental Table 4 for examples of treatment activities).		
Home-based or natural environment treatment delivery: therapy provided in the child’s home or other natural environments to promote generalization and maintenance. Examples: childcare or early intervention center, relatives’ home, rented furnished apartment or extended-stay hotel, or specially created therapy clinic settings. Both indoor and outdoor settings are used.		
Therapy activities are age appropriate, play based, practically useful, and include unilateral, bilateral, and whole-body activities for all groups. UE activities include varied sensorimotor exploration, play with toys and everyday objects, social interactions using gestures and hand games, self-help skills (eating, drinking, washing, dressing), and activities of high interest/novelty for individual infant/toddlers—both indoors and outdoors. Whole-body activities include rolling over, scooting/crawling, pull-to-stand, sitting steadily, walking, bilateral carrying, and large ball play.		
Parent-therapist partnership and home practice component: Parents help identify therapy goals plus implement an additional 45–60 min of daily therapy (at least 5 d/wk) based on child progress during therapist-provided sessions. Therapists provide parents with guidance and updated suggestions through frequent dialogue. Amounts of practice were documented daily by a log the parents kept (in addition to the 3-h therapist-guided sessions).		
Documentation of therapy (systematic data to guide daily sessions): Therapists maintain systematic therapy session logs and provide weekly videotaped sessions that are centrally scored for fidelity of treatment. Parents report to therapists their home component activities. Jointly, therapists and parents review the child’s progress to modify/add treatment goals and activities when appropriate.		
Transfer package to promote future progress: Therapists and parents develop a practical written plan to maintain and progress the child’s UE and other neuromotor skills in everyday and special activities, informed by data about natural developmental progress, and to support engagement in a wide array of age-appropriate activities.		

Abbreviations: CHAMP, Children with Hemiparesis Arm-and-Hand Movement Project; CIMT, constraint-induced movement therapy; OT, occupational therapist; PT, physical therapist; UE, upper extremity.

**TABLE 2.** Baseline Characteristics of Baby CHAMP Study Sample by Treatment Group

Variable	Bimanual/No Constraint, N = 16	CIMT/Part-Time Splint, N = 20	CIMT/Full-Time Cast, N = 19

Age at baseline, mean (SD), mo	18.2 (4.1)	15.6 (5.4)	18.2 (4.1)
Gestational age, mean (SD), wk	38.5 (3.0)	38.2 (3.2)	37.9 (4.2)
Birth weight, mean (SD), oz	114 (27)	106 (35)	103 (28)
Child race (parent report), n			
White	13	20	17
Black/African American	1	0	1
American Indian or Pacific Islander	1	0	0
>1 race	1	0	0
Not reported	0	0	1
Child ethnicity (parent reported), n			
Hispanic	0	1	0
Non-Hispanic	15	16	15
Not reported	0	1	0
Child sex, n			
Boy	5	5	7
Girl	11	15	12
Age when diagnosed with unilateral CP, mean (SD), wk	8.3 (4.0)	8.3 (4.0)	8.4 (4.2)
Side of paresis, n			
Left	5	9	6
Right	11	11	13
Therapy amounts, mean (SD), min/wk			
Occupational therapy	34.7 (23.8)	26.1 (32.6)	52.6 (41.2)
Physical therapy	51.2 (70.0)	41.1 (33.9)	71.7 (44.9)
Baseline Mini-AHA logit scores, mean (SD)	48.6 (12.5)	28.0 (21.0)	27.4 (18.4)
Baseline Bayley-III Fine-Motor paretic side raw scores, mean (SD)	21 (7.9)	14.8 (7.8)	15.6 (8.7)
Baseline Bayley-III Fine-Motor nonparetic side raw scores, mean (SD)	27.2 (8.5)	27.0 (7.4)	24.9 (8.5)

Abbreviations: AHA, Assisting Hand Assessment; Bayley-III, Bayley Scales of Infant and Toddler Development, Third Edition; CHAMP, Children with Hemiparesis Arm-and-Hand Movement Project; CIMT, constraint-induced movement therapy; CP, cerebral palsy.

## WHAT’S KNOWN ON THIS SUBJECT:

Constraint-induced movement therapy and bimanual therapy (hand-arm bimanual intensive therapy [HABIT]) are highly recommended for children aged older than 2 years with unilateral cerebral palsy; for children aged younger than 2 years, there is limited evidence to base recommendations. Clinical trials that directly compare interventions are needed.

## WHAT THIS STUDY ADDS:

All 3 forms of therapist-delivered interventions (20 three-hour sessions in 4 weeks) produced clinically important gains in bimanual and unilateral upper extremity skills, with greatest benefits 6 months posttreatment. Clinicians and families have multiple evidence-based choices to align therapy goals with intervention approaches.

## Abbreviations

AE: adverse event
AHA: Assisting Hand Assessment
Bayley-III: Bayley Scales of Infant and Toddler Development, Third Edition
CHAMP: Children with Hemiparesis Arm-and-Hand Movement Project
CIMT: constraint-induced movement therapy
EoT: end of treatment
FM: fine motor
HABIT: hand-arm bimanual intensive training
IMAL: Infant Motor Activity Log
NIH: National Institutes of Health
PSS-14: 14-item Perceived Stress Scale
RCT: randomized controlled trial
UCP: unilateral cerebral palsy
UE: upper extremity
