# Supplementary material for: Comparative Efficacy RCT of 3 Intensive Infant/Toddler Therapies for Unilateral Cerebral Palsy
Source: Pediatr Open Sci. Author manuscript; Available in PMC 2026 Oct 3. (PMC13631970; doi:10.1542/pedsos.2025-000676)

Supplemental Figure 1.

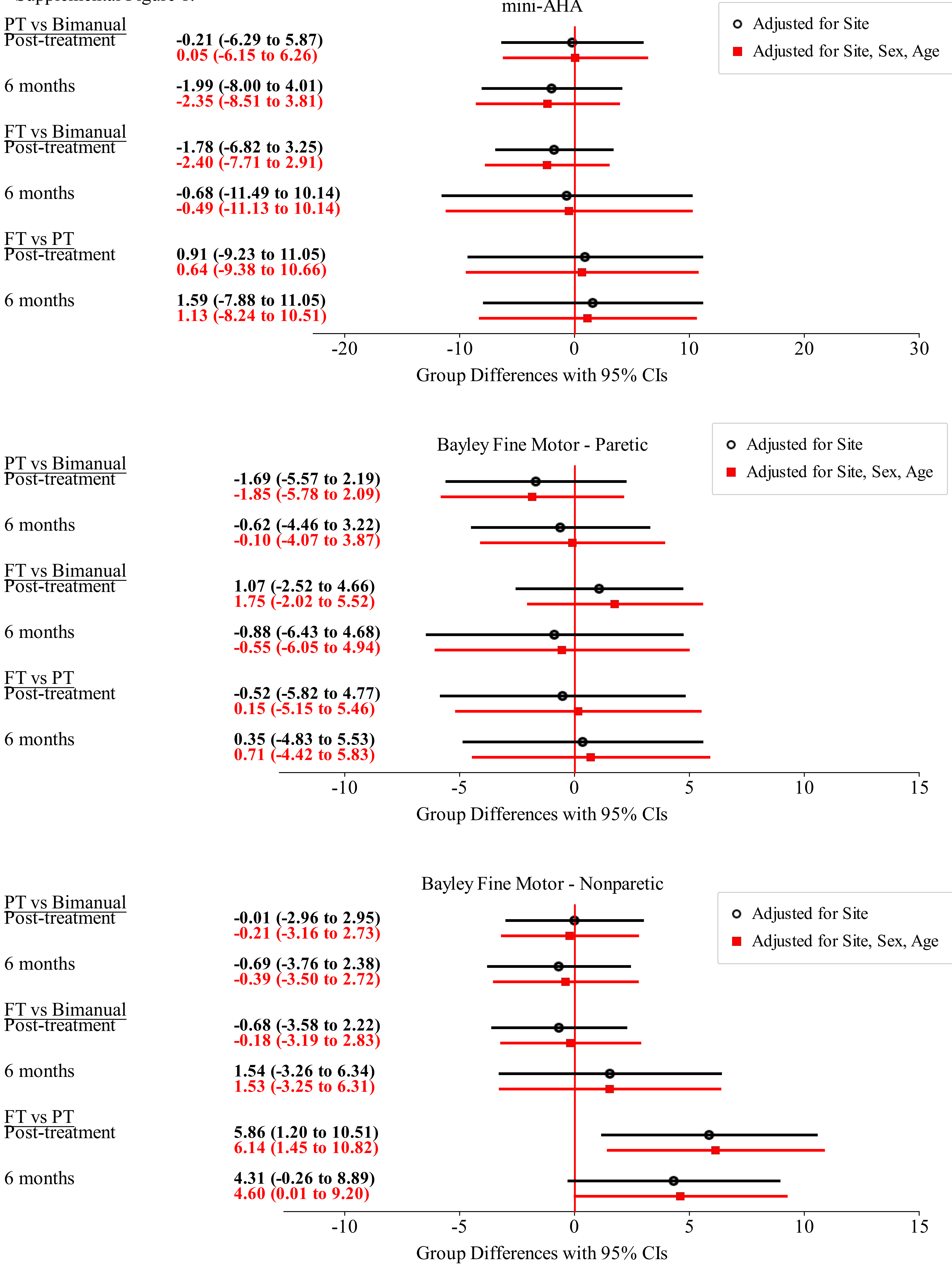

Supplement: Supplementary file 1 [file NIHMS2200058-supplement-Supplemental_1.pdf]
