# Supplementary material for: Comparative Efficacy RCT of 3 Intensive Infant/Toddler Therapies for Unilateral Cerebral Palsy
Source: Pediatr Open Sci. Author manuscript; Available in PMC 2026 Oct 3. (PMC13631970; doi:10.1542/pedsos.2025-000676)

Supplemental Fig. 3. Photos of children showing type of constraint used in the 3 treatment conditions for Baby CHAMP: A. No Constraint   B. Part-time Splint   C. Full-time Cast. For more details for constructing cast and splints see DeLuca SC, et al., 2007, Ramey SL, et al., 2019

A. **B.** **C.**

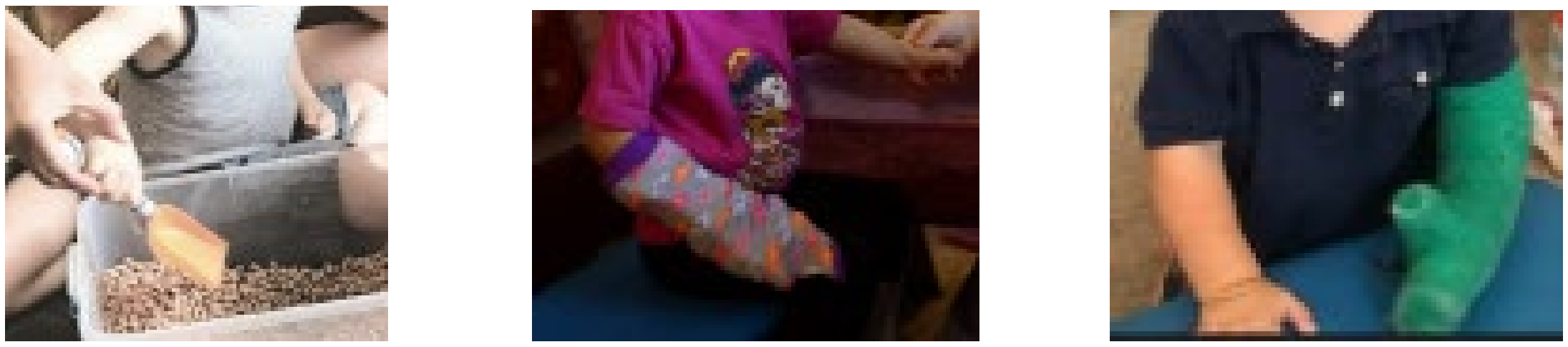

Supplement: Supplementary file 3 [file NIHMS2200058-supplement-Supplemental_3.pdf]
