# Supplementary material for: Comparative Efficacy RCT of 3 Intensive Infant/Toddler Therapies for Unilateral Cerebral Palsy
Source: Pediatr Open Sci. Author manuscript; Available in PMC 2026 Oct 3. (PMC13631970; doi:10.1542/pedsos.2025-000676)
Supplement: Supplementary file 4 [file NIHMS2200058-supplement-Supplemental_4.pdf]

**List of Supplementary Materials**

Figures:
Supplemental Figure 1. Results adjusted for site and sex as covariates.

Supplemental Figure 2. Imputation Results.

Supplemental Figure 3. Photos of children showing type of constraint used in the 3 treatment conditions for Baby CHAMP: A. No Constraint B. Part-time Splint C. Full-time Cast.

Tables:
Supplemental Table 1. Examples of mean differences yielding 80% power for between group differences on the mini-AHA.

Supplemental Table 2. Major and Secondary Outcome Measures by Time of Assessment and Treatment Group. Means reported below are unadjusted for baseline differences, but all statistical analyses control for these differences.

Supplemental Table 3. Analysis of variance tables for the outcomes.

Supplemental Table 4. Examples of treatment activities.

Supplemental Table 1. Examples of mean differences yielding 80% power for between group differences on the mini-AHA.

| CIMT/Part-Time Splint versus Bimanual | CIMT/Full-time Case Splint versus Bimanual |
|---|---|
| 1.0 | 3.45 |
| 1.5 | 3.55 |
| 2.0 | 3.55 |
| 2.5 | 3.44 |
| 3.0 | 3.15 |
| 3.08 | 3.08 |
| 3.5 | 2.30 |
| These are examples of mean differences between groups that yield an effect size (Cohen f = 0.42, $\eta^2$= 0.15) and 80% power with n = 20 participants per group. The power was calculated assuming a within-group standard deviation of 3.46.[23] | |

Supplemental Table 2: Major and Secondary Outcome Measures by Time of Assessment and Treatment Group. Means reported below are unadjusted for baseline differences, but all statistical analyses control for these differences.

| | **Bimanual/No-Constraint** | **CIMT/Part-Time Constraint** | **CIMT/Full-Time Cast** |
|---|---|---|---|
| **Mini-Assisting Hand Assessment Logit Points (scale from 0 – 100)** | | | |
| **Baseline** | | | |
| N | 16 | 20 | 19 |
| Mean (SD) | 48.62 (12.47) | 28.00 (21.00) | 27.42 (18.44) |
| SE | 3.12 | 4.70 | 4.23 |
| Range | 25-66 | 0-64 | 4-72 |
| **End-of-Treatment (EoT)** | | | |
| N | 14 | 18 | 19 |
| Mean (SD) | 52.93 | 33.50 (19.17) | 32.11 (18.24) |
| SE | 15.27 (4.08) | 4.28 | 4.18 |
| Range | 16-69 | 0-63 | 9-77 |
| **6-months Post-Treatment (6-mo)** | | | |
| N | 10 | 11 | 16 |
| Mean (SD) | 55.30 (12.19) | 40.18 (22.27) | 40.75 (22.24) |
| SE | 3.86 | 6.72 | 5.56 |
| Range | 30-72 | 4-66 | 4-82 |
| **Bayley Scales of Infant Motor Development-III Fine-Motor for the Paretic Side (Raw Scores)** | | | |
| **Baseline** | | | |
| N | 16 | 19 | 17 |
| Mean (SD) | 21.00 (7.86) | 14.84 (7.81) | 15.59 (8.70) |
| SE | 1.96 | 1.79 | 2.11 |
| Range | 9-32 | 3-27 | 3-32 |

| | | | |
|---|---|---|---|
| **End-of-Treatment (EoT)** | | | |
| N | 15 | 17 | 18 |
| Mean (SD) | 22.80 (8.26) | 15.29 (8.98) | 17.00 (9.39) |
| SE | 2.13 | 2.18 | 2.21 |
| Range | 9-36 | 6-31 | 1-36 |
| **6-months Post-Treatment (6-mo)** | | | |
| N | 12 | 12 | 16 |
| Mean (SD) | 26.50 (7.66) | 20.92 (10.24) | 20.50 (9.43) |
| SE | 2.21 | 2.96 | 2.36 |
| Range | 9-37 | 6-43 | 7-41 |
| **Bayley Scales of Infant Motor Development-3 Fine-Motor for the Non-Paretic Side (Raw Scores)** | | | |
| **Baseline** | | | |
| N | 16 | 19 | 17 |
| Mean (SD) | 27.19 (8.54) | 26.95 (7.42) | 24.88 (8.51) |
| SE | 2.14 | 1.70 | 2.06 |
| Range | 5-38 | 6-34 | 5-35 |
| **End-of-Treatment (EoT)** | | | |
| N | 15 | 17 | 17 |
| Mean (SD) | 30.07 (5.39) | 28.76 (6.86) | 26.88 (7.53) |
| SE | 1.39 | 1.66 | 1.83 |
| Range | 18-41 | 14-37 | 15-39 |
| **6-months Post-Treatment** | | | |
| N | 12 | 12 | 16 |
| Mean (SD) | 30.92 (8.23) | 31.42 (8.04) | 34.94 (7.55) |
| SE | 2.38 | 2.32 | 1.89 |

| | | | |
|---|---|---|---|
| Range | 16-40 | 18-43 | 14-49 |
| **Infant Motor Activity Log – How Often** | | | |
| **Baseline** | | | |
| N | 15 | 18 | 18 |
| Mean (SD) | 1.62 (0.83) | 0.89 (0.91) | 1.30 (1.16) |
| SE | 0.22 | 0.21 | 0.27 |
| Range | 0.20-3.40 | 0.00-3.50 | 0.05-4.60 |
| **End-of-Treatment (EoT)** | | | |
| N | 15 | 18 | 16 |
| Mean (SD) | 3.19 (0.82) | 2.19 (1.12) | 2.02 (1.18) |
| SE | 0.21 | 0.26 | 0.29 |
| Range | 1.63-4.05 | 0.50-4.50 | 0.40-4.85 |
| **6-months Post-treatment (6-mo)** | | | |
| N | 13 | 10 | 14 |
| Mean (SD) | 3.16 (1.13) | 2.37 (1.35) | 2.30 (1.10) |
| SE | 0.31 | 0.42 | 0.29 |
| Range | 1.45-4.95 | 0.65-4.45 | 0.70-4.25 |
| **Infant Motor Activity Log – How Well** | | | |
| **Baseline** | | | |
| N | 15 | 17 | 18 |
| Mean (SD) | 1.79 (0.98) | 1.00 (0.86) | 1.44 (1.12) |
| SE | 0.25 | 0.21 | 0.26 |
| Range | 0.20-4.10 | 0.10-3.25 | 0.05-4.30 |
| **End-of-Treatment** | | | |
| N | 15 | 18 | 16 |

| | | | |
|---|---|---|---|
| Mean (SD) | 3.19 (0.88) | 2.16 (1.11) | 2.09 (1.15) |
| SE | 0.23 | 0.26 | 0.29 |
| Range (Min-Max) | 1.35-4.10 | 0.45-4.50 | 0.40-4.75 |
| **6-months Post-Treatment** | | | |
| N | 13 | 10 | 14 |
| Mean (SD) | 3.15 (1.23) | 2.39 (1.28) | 2.34 (1.19) |
| SE | 0.34 | 0.40 | 0.32 |
| Range (Min-Max) | 1.15-4.95 | 0.55-4.00 | 0.55-4.00 |

Supplementary Table 3: Repeated Measures Analysis of Covariance (ANCOVA) Model for Outcomes. *significance at p<0.05, ** P<0.001.

| Effect | DF | N | F Statistics | P |
|---|---|---|---|---|
| **The Mini-Assisting Hand Assessment** | | | | |
| Baseline | 1 | 47 | 208.57 | <0.0001** |
| Site | 1 | 47 | 20.02 | 0.1619 |
| Group | 2 | 47 | 0.01 | 0.9874 |
| Time | 1 | 47 | 9.51 | 0.0034* |
| Group X Time Interaction | 2 | 47 | 0.37 | 0.6956 |
| **Bayley Scales of Infant and Toddler Motor Development-III, Fine Motor for the Paretic Side** | | | | |
| Baseline | 1 | 46 | 100.12 | <0.0001* |
| Site | 1 | 46 | 1.90 | 0.1749 |
| Group | 2 | 46 | 0.24 | 0.7898 |
| Time | 1 | 46 | 11.25 | 0.0016* |
| Group X Time Interaction | 2 | 46 | 0.05 | 0.9535 |
| **Bayley Scales of Infant and Toddler Motor Development-III, Fine Motor for the Non-Paretic Side** | | | | |
| Baseline | 1 | 45 | 65.49 | <0.0001** |
| Site | 1 | 45 | 0.93 | 0.3409 |
| Group | 2 | 45 | 1.33 | 0.2744 |
| Time | 1 | 45 | 21.96 | <0.0001** |
| Group X Time Interaction | 2 | 45 | 5.36 | 0.0083* |

Supplemental Table 4. Examples of treatment activities.

| Group 1<br>CIMT with Full-Time Cast | Group 2<br>CIMT with Part-Time Splint | Group 3<br>Bimanual/No Constraint |
|---|---|---|
| Continuous wearing of full-arm lightweight cast for first 17 of 20 therapy sessions. Constraint removed for final 3 days when therapy focus is on integrating new UE skills into bimanual activities. | Part-time use of part-arm splint worn only during first 17 therapy sessions. No constraint in final 3 days when therapy focus is on integrating new UE skills into bimanual activities. | No constraint is used during and in-between formal treatment sessions. |
| **Examples of Activities Common Across Groups** | | |
| Unilateral Activities | | |
| Self-Feeding - Finger feeding, Holding handled cups/bottles, infant utensils | | |
| Pushing button to activate musical or light up toys | | |
| Grasp and release of palm-size objects | | |
| Puzzle piece placement | | |
| Scooping with play shovel | | |
| Bilateral and whole Body Activities | | |
| Crawling, 4-point quad weight bearing | | |
| Holding, rolling a large ball | | |
| Pushing toy cart or handled wheeled toy | | |
| Cruising (e.g., using arms to hold to furniture, bar to walk alongside) | | |
